# Endoscopic hybrid treatment for refractory duodenal varices using endoscopic ultrasound–guided coil deployment and cyanoacrylate injection

**DOI:** 10.1055/a-2876-1772

**Published:** 2026-06-23

**Authors:** Hirotsugu Maruyama, Atsushi Irisawa, Yoshinori Shimamoto, Yuji Kawata, Kojiro Tanoue, Akira Yamamoto, Yasuhiro Fujiwara

**Affiliations:** 1 Department of Gastroenterology 12935 Osaka Metropolitan University Graduate School of Medicine School of MedicineOsakaOsaka PrefectureJapan; 2 Department of Gastroenterology 175771 Dokkyo Medical University School of MedicineShimotsuga DistrictTochigi PrefectureJapan; 3 Gastroenterology 12935 Osaka Metropolitan University, Graduate School of MedicineOsakaOsaka PrefectureJapan; 4 Department of Diagnostic and Interventional Radiology 12935 Osaka Metropolitan University Graduate School of MedicineOsakaOsaka PrefectureJapan

**Keywords:** Endoscopic ultrasonography

No standard treatment has been established for duodenal varices; management involves a combination of endoscopic therapy 1​ and interventional radiology (IVR) procedures, such as balloon-occluded retrograde transvenous obliteration or percutaneous transhepatic obliteration. 2​ In refractory cases or when IVR is not feasible, options are limited, but endoscopic ultrasound-guided variceal therapy has recently emerged as a promising alternative. 3​ We report a refractory recurrent duodenal varix, unsuitable for IVR due to portal vein stenosis, successfully treated with hybrid endoscopic therapy.

A 21-year-old man with idiopathic portal hypertension was admitted for worsening duodenal varices ( Fig. 1 ). He had previously undergone partial splenic embolization (PSE) and endoscopic cyanoacrylate injection; however, varices recurred ( Fig. 2 ). Given the patient’s young age, need for definitive cure, and risk of overwhelming postsplenectomy infection, minimally invasive endoscopic treatment was selected over PSE or splenectomy. First, endoscopic ultrasound-guided coil deployment was performed. A 22 G needle (EZshot3 plus; Olympus Medical Systems, Tokyo, Japan) was used to puncture the drainage side, and after confirming intravascular placement, a 10 mm×32 cm AZUR HydroCoil (Terumo Corporation, Tokyo, Japan) was deployed. Coil placement was confirmed by computed tomography (CT), after which cyanoacrylate was injected into the duodenal varices under direct visualization using upper gastrointestinal endoscopy ( Video 1 ). No complications occurred, and CT demonstrated adequate treatment, with no migration of cyanoacrylate into the portal vein ( Fig. 3 ).

**Fig. 1:**
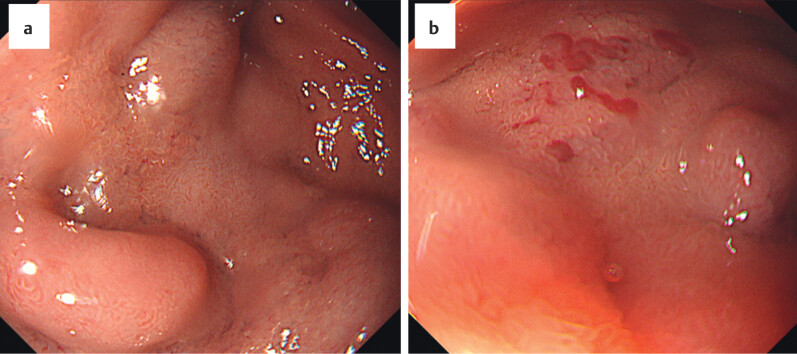
Esophagogastroduodenoscopic findings. ( **a** ) Duodenal varices (F1–F2) were identified in the duodenal bulb. ( **b** ) Red color signs were observed on the varices.

**Fig. 2:**
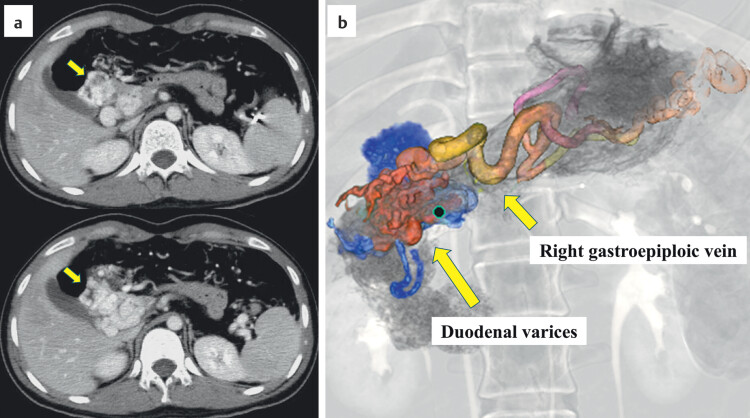
Contrast-enhanced computed tomography (CT) and 3D-CT findings. ( **a** ) Duodenal varices were observed on contrast-enhanced CT (portal venous phase; yellow arrows). ( **b** ) Three-dimensional CT demonstrated that the duodenal varices had developed with the right gastroepiploic vein as the feeding vessel.

**Video 1** Endoscopic hybrid treatment for refractory duodenal varices using endoscopic ultrasound–guided coil deployment and cyanoacrylate injection.


**Fig. 3:**
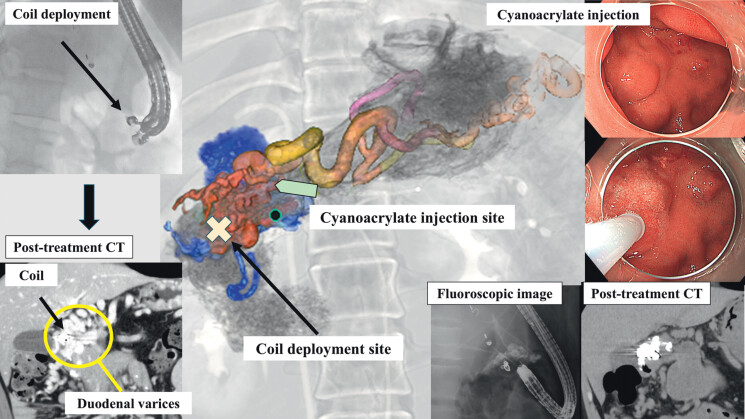
Endoscopic hybrid treatment. In this treatment, endoscopic ultrasound–guided coil deployment was initially performed. Subsequently, contrast-enhanced computed tomography (CT) was used to confirm the position of the coil and the presence of residual duodenal varices. Upper gastrointestinal endoscopy was then performed to visualize the varices on the luminal side of the duodenum, followed by cyanoacrylate injection. Adequate thrombosis over a sufficient area was confirmed on fluoroscopic imaging and post-procedural CT, indicating that appropriate endoscopic therapy had been achieved.

This method is particularly effective for ectopic varices exhibiting complex hemodynamics, such as duodenal varices. If the hemodynamics, including the inflow and outflow pathways, can be identified through various preliminary tests, we believe that placing a coil on the outflow side and injecting cyanoacrylate into the target variceal from within the gastrointestinal lumen will yield significant results. It enables the treatment of a wide range of varices and provides a therapeutic option when IVR is not feasible.

Endoscopy_UCTN_Code_TTT_1AS_2AL

## References

[1] Chu J D Bi Q Wang Y L Duodenal varices and evaluation of endoscopic cyanoacrylate injection for treatment of duodenal variceal bleeding ILIVER 2025 17 100143 10.1016/j.iliver.2025.100143 PMC12212683 40636782

[2] Dzwonkowski M Iqbal U Kaufer S W Endoscopic band ligation of bleeding duodenal varices Cureus 2022 14 e22009 10.7759/cureus.22009 35340508 PMC8913514

[3] Masuda S Irisawa A Nakaya S Endoscopic ultrasonography-guided variceal therapy as salvage treatment for rebleeding from duodenal varices following balloon-occluded retrograde transvenous obliteration DEN Open 2025 6 e70183 10.1002/deo2.70183 40821723 PMC12352126

